# Optimized Mouse Model for the Imaging of Tumor Metastasis upon Experimental Therapy

**DOI:** 10.1371/journal.pone.0026810

**Published:** 2011-11-03

**Authors:** Sergio Lavilla-Alonso, Usama Abo-Ramadan, Juha Halavaara, Sophie Escutenaire, Turgut Tatlisumak, Kalle Saksela, Anna Kanerva, Akseli Hemminki, Sari Pesonen

**Affiliations:** 1 Cancer Gene Therapy Group, Molecular Cancer Biology Program, Transplantation Laboratory, Haartman Institute and Finnish Institute of Molecular Medicine, University of Helsinki, Helsinki, Finland; 2 HUSLAB, Helsinki University Central Hospital, Helsinki, Finland; 3 Department of Neurology, Helsinki University Central Hospital, Helsinki, Finland; 4 Department of Radiology, Helsinki University Central Hospital, Helsinki, Finland; 5 Department of Radiology, Jorvi Hospital, Espoo, Finland; 6 Department of Virology, Haartman Institute, University of Helsinki and Helsinki University Central Hospital, Helsinki, Finland; 7 Department of Obstetrics and Gynecology, Helsinki University Central Hospital, Helsinki, Finland; Stanford University, United States of America

## Abstract

Development of new cancer treatments focuses increasingly on the relation of cancer tissue with its microenvironment. A major obstacle for the development of new anti-cancer therapies has been the lack of relevant animal models that would reproduce all the events involved in disease progression from the early-stage primary tumor until the development of mature metastatic tissue. To this end, we have developed a readily imageable mouse model of colorectal cancer featuring highly reproducible formation of spontaneous liver metastases derived from intrasplenic primary tumors. We optimized several experimental variables, and found that the correct choice of cell line and the genetic background, as well as the age of the recipient mice, were critical for establishing a useful model system. Among a panel of colorectal cancer cell lines tested, the epithelial carcinoma HT29 line was found to be the most suitable in terms of producing homogeneous tumor growth and metastases. In our hands, SCID mice at the age of 125 days or older were the most suitable in supporting consistent HT29 tumor growth after splenic implantation followed by reproducible metastasis to the liver. A magnetic resonance imaging (MRI) protocol was optimized for use with this mouse model, and demonstrated to be a powerful method for analyzing the antitumor effects of an experimental therapy. Specifically, we used this system to with success to verify by MRI monitoring the efficacy of an intrasplenically administered oncolytic adenovirus therapy in reducing visceral tumor load and development of liver metastases. In summary, we have developed a highly optimized mouse model for liver metastasis of colorectal cancer, which allows detection of the tumor load at the whole body level and enables an accurate timing of therapeutic interventions to target different stages of cancer progression and metastatic development.

## Introduction

Uncontrolled progression of primary tumors is a usual cause of cancer-related deaths, especially in colorectal cancer. For this reason, in the last decades cancer research focuses, not only on the genetic causes of cancer, but on epigenetic variables and influence of tumor microenvironment. Phenomena like tumor invasiveness, progression and metastases need to be understood in order to find more efficient treatment modalities. However, a big demand exists for more realistic and reliable metastatic models that feature all phases of the metastatic process.

For basic research on metastasis and development of new anticancer agents, the selection of the optimal animal model is of great importance and is dependent on which biological questions are addressed. Subcutaneous xenograft murine models are often used since such tumors are easy to grow, and the tumor size and treatment responses can be followed non invasively. However, such models are not optimal for all purposes, as the subcutaneous tumor environment may not fully resemble relevant clinical situation such as liver metastases in the context of advanced colorectal cancer [1]. It is increasingly recognized that the tumor environment plays a key role in the behavior of the tumor and the response to therapeutics [2]. In this regard, subcutaneous tumors are problematic, as the growth environment is different from naturally occurring tumors, making their morphology and especially stromal elements markedly different [3]. Most importantly, subcutaneous tumors are poorly invasive rarely lead to metastases. This is a major disadvantage since majority of overall cancer related deaths are due to metastases. Therefore, detection, prevention, and treatment of distant metastases have become increasingly important objectives in cancer research.

Fortunately, metastasis is a rare event. Specific conditions are needed for disseminating cells to develop metastasis, and only selected cells detached from their primary site are capable of establishing a mature tumor in a distant ectopic location, given that the appropriate conditions are available [4]. Even when metastatic cells have reached a recipient organ, they may remain as single cells or as micrometastases until certain factors permit their development to metastases [5]. Metastasis is a multistage process including cell migration, entry into vasculature, survival in the circulation/lymphatic vessels, invasion of vessel wall, migration to target organ, implantation, and tumor formation at the metastatic site [6] . The whole process may take several years or even decades [6]. Such complexity makes it difficult to study metastatic cancers, and most in vivo models only reproduce some of the aforementioned steps.

For the purpose of research and therapeutic development, some metastatic animal models are available [7]. In many of them, cells are injected directly to the systemic circulation and the site of metastasis is mostly defined by the first capillary bed the tumor cells meet after injection and, in a lesser extent, influenced by cancer cell type [7]. For example, after tail vein injection tumors are found in the lungs, while after intracardiac injection, tumors can be found at several sites [7]. In this type of experimental models, early steps of the metastatic process are not recapitulated, which may lead to differences in tumor behavior when compared to metastases deriving from a primary tumor [8].

Several genetically engineered mouse strains producing metastatic disease bona fide are available [9]. The tumors arising in these models are, however, fully of murine origin, and may not accurately represent the properties of human tumors, nor do they allow to study certain human specific therapeutic agents, such as oncolytic adenoviruses.

Magnetic Resonance Imaging (MRI) is a powerful imaging modality for characterizing animal models of disease. Contrast-enhanced MRI has significantly improved the accuracy of the detection of several tumors, such as brain and liver tumors. Tissue-specific contrast agents such as superparamagnetic iron oxide (SPIO) have been reported to enable highly sensitive to the detection of liver tumors [10].

Based on systematic testing of different colorectal cancer cell lines and mouse backgrounds, we describe here a new colorectal cancer mouse model featuring spontaneous liver metastases originating from an intrasplenic primary tumor. In this mouse model, MRI allowed effective in vivo monitoring of tumor growth and treatment response which permitted a variety of longitudinal studies not possible with invasive methods. For example, repeated measurements of the same animal were possible thus reducing the total number of animals needed in the study. The sensitivity of the model for detecting the effects of antitumor drugs was demonstrated by using oncolytic adenovirus as an example of a novel therapeutic agent.

## Results

### Selection of mice strain and provider

**Figure 1:**
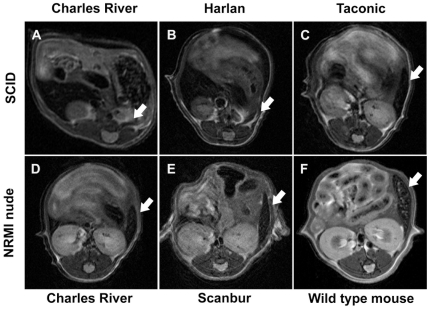
Developmental stage of the spleens of adult SCID and NMRI nude mice. 125 to 135 days old SCID and NMRI nude mice from different providers were imaged with MRI to evaluate the developmental stage of the spleens (arrows). For SCID mice, animals purchased from Charles River (A) and Harlan (B) showed poorly developed, diffuse, and small spleens while animals from Taconic (C) had solid, compact, and relatively large spleens. NMRI nude mice from Charles River (D) and Scanbur (E) showed fully developed spleens with a similar appearance.

In order to identify an optimal mouse strain to support our model, athymic NMRI nude and SCID mice were tested. For successful implantation and proper growth of intrasplenic tumors, the consistency, size, and structure of the spleen were found to be of critical importance. In general, mutations used to generate deficiencies of the immune system, such as found in nude and SCID mice, truncate normal spleen development [11] and lead to morphologically immature spleens in adult mice (Figure 1). Interestingly, important differences in spleen morphology were seen in MRI analysis between mice strains. As a curiosity, variation in the splenic structure was also seen among mice with the same genetic defect (SCID) but from a different provider.

As confirmed by morphological observation after laparotomy, NMRI nude mice had a solid, compact and relatively large spleen. In contrast, SCID mice of the same age often presented an immature spleen: the organ was fragile, small, soft, and pale in comparison to wild type (NMRI) mice. This was also observed with MRI (Figure 1) where the spleens of SCID mice not only appear smaller than seen in wild type (NMRI) mice, but are squeezed between neighboring organs due to their diffuse/malleable structure. In comparison to SCID mice, nude mice under anesthesia were more susceptible to hypothermia during the imaging session and were not therefore used for optimization of the MRI method. To further evaluate the spleen development of SCID mice, animals were imaged at the age of 125 days or older. All animals obtained from Taconic had mature splenic tissue at the time of imaging (Figure 1) and were therefore selected for further experiments.

After cancer cell injection (HT29 cells), only mice with mature splenic tissue showed proper tumor engraftment (Table 1). Animals with immature spleens showed no intrasplenic tumor development, and, when present, tumors were most often localized in the peritoneal cavity or in tissues adjacent to the spleen, suggesting spillage of injected. In contrast, 20 out of 20 SCID mice from Taconic presented detectable tumors 21 days after cancer cell implantation, indicating spontaneous origin from the primary splenic tumor. For NMRI nude mice, 3 out of 7 (Charles River) and 2 out of 4 (Scanbur) animals showed intrasplenic tumor development.

**Table 1:** Morphology of the spleens of 30- and 125-day-old SCID and NMRI nude mice.

Mouse strain	Provider	Suitable for IS injection at age of30 days	Suitable for IS injection at age of 125 days	Mice developing intrasplenic tumor (ratio)	Mice developing hepatic metastases (ratio)
**SCID**	Charles River (Sulzfeld, Germany)	No	No	NA	NA
**SCID**	Harlan (Horst, Holland)	No	No	NA	NA
**SCID**	Taconic (Lille Skensved, Denmark)	No	Yes	20/20 (100%)	20/20 (100%)
**NMRI-nu/nu**	Charles River (Sulzfeld, Germany)	Yes	Yes	3/7 (43%)	3/7 (43%)
**NMRI-nu/nu**	Scanbur (Sollentuna, Sweden)	Yes	Yes	2/4 (50%)	2/4 (50%)

SCID, severe combined immunodeficiency; NA, not applicable.

With regard to liver metastases originating from the primary spleen tumor, SCID mice presented hepatic tumors in 100% of the mice used. These tumors were first seen with MRI one week after the primary tumor detection.

### Selection of a human colorectal cancer cell line

**Figure 2:**
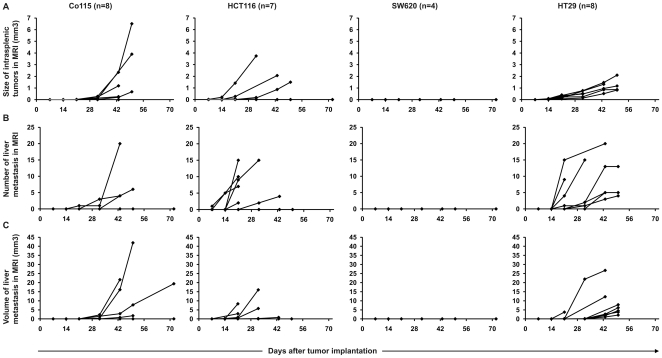
Development of intrasplenic and intrahepatic tumors in SCID mice. Four human colorectal cancer cell lines (Co115, HCT116, SW620, HT29) were tested for their ability to induce growth of intrasplenic and intrahepatic tumors in SCID mice. 1x10e6 cells were injected into the spleen and primary tumor growth (A), number of metastasis in the liver (B), and total volume of the liver tumors (C) were analyzed weekly by MRI. Each line represents an individual animal. Number of animals used for testing each cell line is presented in parentheses.

A panel of human colorectal cancer cell lines (Co115, HCT116, SW620, HT29) was compared in SCID mice for their ability to induce intrasplenic and intrahepatic tumors after cancer cell implantation in the spleen (Figure 2). Co115 cells produced intrasplenic and intrahepatic tumors in 5/8 and 3/8 animals, respectively, between 14 and 43 days after cell injection. For HCT116 cells, intrasplenic and intrahepatic tumors appeared in 5/7 and 6/7 animals, respectively, between 7 and 32 after cell implantation. SW620 did not induce tumors neither in the spleen nor liver in any of the animals (0/4). For HT29, all animals (8/8) developed both primary tumors and metastases. In all cases, intrasplenic tumor growth was detectable by 21 days after cell injection while tumors in the liver were first observed 1 to 4 weeks later, starting from day 28 after cell implantation. Based on these results the HT29 cell line was considered to be superior compared to Co115, HCT116 and SW620, and was thus selected for further studies.

### Metastatic origin of the hepatic tumors

The portal system allows easy spread of metastatic cells from splenic tumors to the liver but may also mediate direct implantation of the cells in hepatic tissue after intrasplenic injection. Therefore, it is crucial to distinguish between these events. The development of hepatic tumors in the absence of tumors in the spleen would be assumed to be a consequence of a direct implantation of the injected cells in the liver. This was seen in mice injected intrasplenically with the cell line HCT116. On day 21 after intrasplenic cancer cell injection, 2 out of 7 mice presented hepatic tumors but no intrasplenic tumors and 1 mouse showed detectable intrasplenic tumor several days after appearance of the intrahepatic tumor. On the contrary, for all mice injected with HT29 cells, the intrahepatic liver tumors developed 7 to 21 days after the appearance of primary intrasplenic tumors, strongly supporting the metastatic origin of these tumors.

### MRI method optimization

Current MRI methodology in small animals is not readily applicable for testing large number of subjects in a short time, because it would increase costs and would require to keep the animals under anaesthesia for too long. Therefore, our MRI method was optimized in order to achieve the following goals: 1) Fast and accurate detection of liver tumors 2) minimized imaging time in order to be able to image a large amount of animals within a reasonable time; and 3) minimal time under anaesthesia to avoid associated health risks. In order to achieve two latter goals, we designed an MRI protocol with an imaging time of around 10 minutes with a requirement for the mice to be under isoflurane anaesthesia for less than 30 minutes. This schedule was well tolerated and no mortality was seen even upon repeated imaging. For optimal visualization of liver tumors, we chose to assist imaging with systemic administration of Endorem (Roissy CdG Cedex, France), an SPIO contrast agent that accumulates in the Kupffer cells of the liver. Its predominant action is to shorten the T2 or T2* relaxation time by disturbing the local magnetic field in the liver parenchyma. The signal intensity from normal liver dramatically decreases after SPIO administration. While malignant liver foci do not contain Kupffer cells, their signal remains unaltered, and the signal difference between liver and tumor lesions increases.

In our work, liver signal was reduced to 32% and 25% after 15 and 45 minutes from contrast agent administration, respectively. At later time points, signal remained at a constant 19% compared to non-treated livers. To maximize the lesion–liver contrast, all tests were performed at least 30 minutes after contrast agent administration.

The stable decrease of liver signal from 15 minutes until late timepoints by Endorem administration optimized the quality of the images in a minimal analysis time. In addition, an imaging time of only 10 minutes reduced dramatically the possibility of animals dying of hypothermia in the MRI device. With this improved MRI method, each animal could be fully imaged only in 30 minutes. This increases the performance of the method, reduces number of mice needed and permits screening of large number of animals in short periods of time, decreasing costs.

### Use of the optimized mouse model to study the effects of an experimental treatment

**Figure 3:**
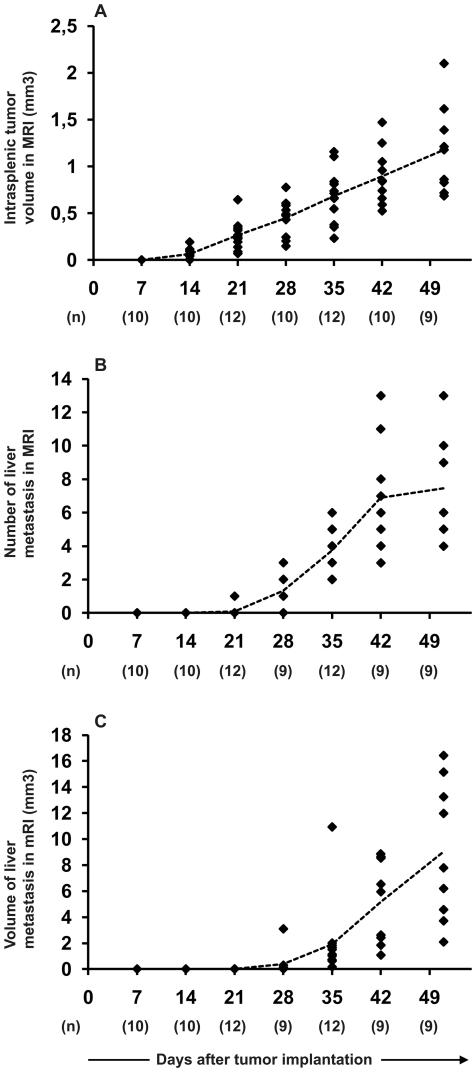
Validation of HT29 cell line in the metastatic colorectal cancer model. 1x10e6 cells were injected into the spleen of 125-day-old SCID mice and intrasplenic tumor growth (A), number of metastatic lesions in the liver (B), and total volume of liver metastases (C) were assessed weekly with MRI. Each dot represents an individual animal and mean of each time point is marked with dotted line. Number of animals analyzed at each time point is presented in parentheses.

Animals were implanted with HT29 cells and imaged with MRI weekly for 6 weeks. As expected, mice developed both intrasplenic tumors and liver metastases (Figure 3). As also verified before, liver metastases were detected in each case 1 to 3 weeks after intrasplenic (primary) tumor formation. At least one week before metastases were detected, primary tumors were considered suitable for an 8 µl microinjection of the therapeutic agent. Of note, this time window of at least one week makes the model useful for the evaluation of possible pro- and anti-metastatic properties as well as the intervention, in this case the viral injection.

**Figure 4:**
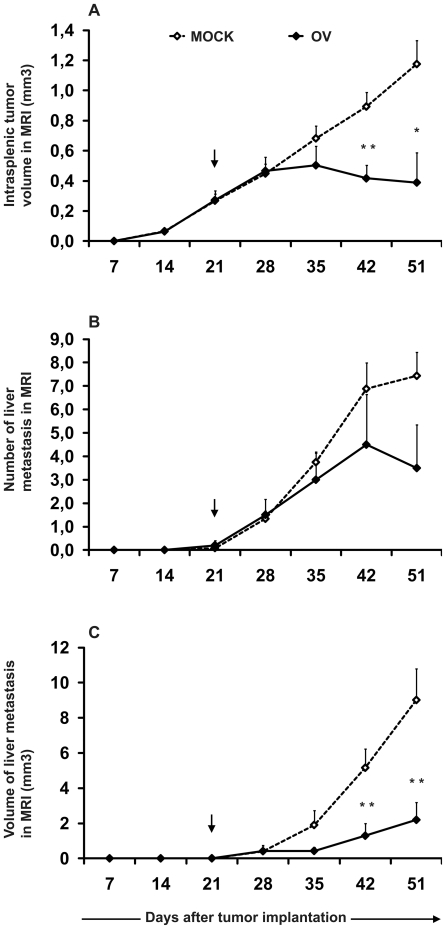
MRI based evaluation of the effect of oncolytic adenovirus Ad5-D24-RGD in metastatic colorectal cancer mouse model. 1x10e6 HT29 cells were injected into the spleen of 125-day-old SCID mice. Intratumoral injection of the virus (OV) at a dose of 2x10e7 viral particles was given 21 days later (arrow). Intrasplenic tumor growth (A), number of metastatic lesions in the liver (B) and a total volume of liver metastasis (C) were assessed weekly with MRI. Data is presented as mean ± SD. *, p<0.05, **, p<0.01.

Adequate sensitivity to detect changes in the visceral tumor load is a critical requirement for a model to be useful in the evaluation of the potency of therapeutic agents for orthotopic colorectal cancer. Oncolytic adenoviruses have been widely tested in mouse xenograft models of human cancers and shown to effectively reduce tumor masses [12]. Here, we used an oncolytic adenovirus Ad5-D24-RGD as an example of a developmental anti-cancer drug to assess model utility for quantifying treatment responses. Significant intrasplenic tumor growth reduction, detected after a latency period of two weeks (i.e. days 21 to 35 after tumor implantation), was seen following a single intratumoral administration of a moderate dose of oncolytic adenovirus (Figure 4A). In contrast to untreated mice, whose tumors grew steadily until the end of the experiment, tumors of treated animals stopped growing. Intratumoral treatment of primary spleen tumors with oncolytic adenovirus did not have a pro-metastatic effect. This was seen both as a reduced number of metastatic lesions (Figure 4B) as well as significantly smaller amount of total tumor mass in the liver (Figure 4C).

### Comparison of tumors in laparatomy and MRI

**Figure 5:**
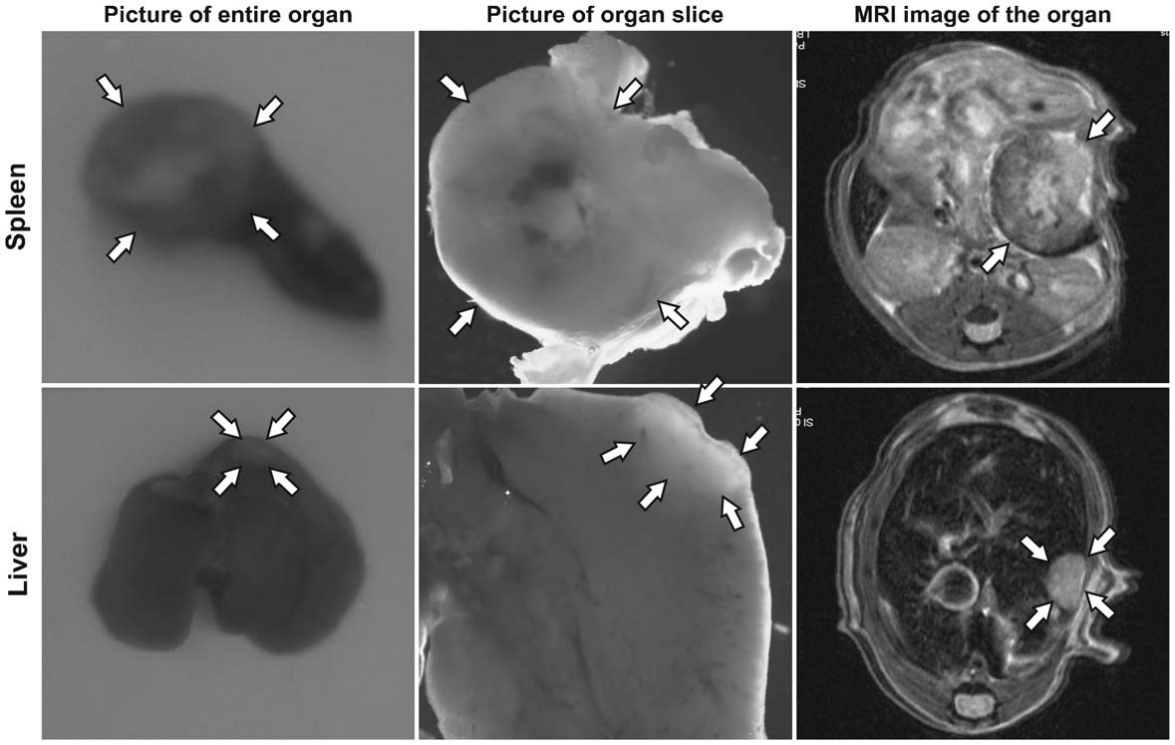
Visualization of primary tumor in the spleen and liver metastasis. Laparotomy observations of the entire organs and sections of the organs where the tumors are engrafted give an accurate idea of the location and shape of the spleen and liver tumors within the organ, which corresponds to its predicted location and shape by MRI.

For its optimal utility, the imaging method should allow fast and progressive identification of tumor development in the spleen and the liver. A general difficulty with MRI is to differ between tumors and stromal elements such as vessels, the gall bladder, hepatic lymph nodes or ligaments. Therefore it was important to compare our MRI results with laparotomy findings. As shown in figure 5, laparotomy observations of the entire organ (5A) and of a section of the organ where the tumor is engrafted (5B) give an accurate idea of the location and shape of the tumor within the organ, which corresponds to its predicted location and shape by MRI (5C). This demonstrates that MRI is a reliable method for splenic and liver tumors diagnosis in mice.

## Discussion

Here we describe the development and use of an extensively optimized liver metastatic colorectal cancer mouse model, which provides several important advantages compared to other metastatic mouse models described before. Moreover, the utility of MRI for monitoring changes in tumor growth and rate of metastasis in that model was optimized and demonstrated by using oncolytic adenovirus Ad5-D24-RGD as a model treatment agent.

Four colorectal cancer cell lines were tested and remarkable differences in primary tumor growth rates and metastasis formation were observed. Epithelial HT29 cell line showed the best reproducibility both in primary tumor and metastasis development. 100% (20/20) of animals showed growth of intrasplenic tumors within 2 weeks from cell transplantation, and liver metastases were detected in all animals 1 to 3 weeks afterwards. This suggests a metastatic nature for the HT29 liver tumors rather than being a consequence of direct immediate dissemination from the spleen through the portal vein, as has been reported for other colorectal cancer cell lines [13]. If the liver tumors would have been a consequence of accidental direct injection into the portal vein, they would have been expected to appear concurrently with spleen tumors. In comparison to other colorectal cancer cell lines, the growth rate of HT29 tumors was relatively slow and uniform, which might be an advantage with regard to flexibility of experimental design. Also, slower growth would correspond better with the typical behavior of human tumors. All animals were killed due to progressing metastatic disease 6 to 7 weeks from the beginning of the experiment.

Spleen was used as a primary target organ for tumor cell implantation in our model. It is known that the mutations that render laboratory mice immune deficient, can result also in developmental defects in their spleens [11], and the developmental stage of the spleen at the time of tumor cell injection had a significant effect on the outcome of cell implantation. Immature spleens did not allow tumor engraftment at the injection site and consequently tumors were often found in the peritoneal cavity or in tissues adjacent to the spleen, suggesting direct extravasation from the injection site. Based on our observations, differences in the developmental stage of spleens may significantly alter the outcome of tumor cell injection, which is of critical importance for reduction of the biological variation in tumor and metastasis development. Therefore, anatomical confirmation of the proper structure of the spleens prior to cancer cell implantation (with e.g. MRI) is crucial in order to ensure uniform development of liver metastases.

Metastatic progression is one of the most severe aspects in cancer. The metastatic process not only depends on the characteristics of the primary tumor cells, but also on the tumor microenvironment, where extreme conditions such as poor oxygenation and nutrition, high interstitial pressure, presence of immune cells, extracellular matrix and isolation from the host organ by stromal barriers are often present [14]–[16]. Metastatic development also depends on the target tissue and the capability of its stromal cells to give rise to a metastatic tumor [15], [17]. Liver is the primary target organ for metastases in colorectal cancers [6], [18]. To develop clinically relevant models, different approaches have been utilized for human cancer cell engraftment in the murine liver. Direct injection of cancer cells either in the liver parenchyma [19] or in the portal vein [20] has been used to induce tumors in the liver. Neither approach allows for selection of metastatic cells from injected cells. This may partially explain the variability often seen in the number of mice developing intrahepatic tumors in this type of experimental set-up [13], [19], [20]. Also, implantation of a primary tumor (individual cells or solid tumors) into spleen [13] or cecum [21] for later release of disseminated cells to the portal system has been described earlier. Main disadvantage of these models is that only a portion of animals develop disseminated tumors and/or the location of primary or metastatic tumors vary increasing the number of animals needed in each experiment.

It has been shown that metastatic cells detaching from the spleen are assisted by splenocytes on their way to the liver, demonstrating the involvement of microenvironmental factors in the metastatic process also in mice [13]. As supportive evidence for the importance of metastatic selection of the cells, more uniform tumor development was achieved if the cells were implanted in the spleen (vascular access to the liver though the portal system) in comparison to direct injection of the same cell line into the portal vein [13]. In our model, spleen was selected as the most suitable injection site since it is easily accessible with low-mortality surgery (0% in our experiments). In addition, we propose the portal system as the most relevant route for allowing cells to disseminate into the liver, since it mimics the clinical situation most closely. However, the absence of immune system should be considered as a common limitation in all animal models utilizing cancer cells from human origin. The role of immune cells in the formation of true tumor microenvironment is omitted in all murine models of human cancer.

The progression of liver metastatic disease cannot be followed up by external observation of the object. Overall symptoms do not linearly correlate with the progression rate and only when critical tumor mass is achieved (approx. 1kg in humans) the patient starts to show obvious signs of disease. Therefore, a model capable of assessing the dissemination of disease in its early stages in the absence of external symptoms is useful for developing new modalities for the prevention and treatment of metastatic colorectal cancer. The spleen-to-liver colorectal cancer model described here seems to meet these requirements and might be suitable as such a model.

Animal models utilizing viscerally implanted orthotopic tumors may be more clinically relevant than subcutaneous models, because they incorporate the effects of the tissue environment better, but tumors can usually be measured only after death which might lead to loss of data at potentially relevant early time points. Thus, real-time monitoring would be optimally required to evaluate the effects of treatments on metastases. Here, we validated an MRI method to monitor intrasplenic and intrahepatic tumor volumes over time. To enhance contrast between tumor and non-tumor tissue, a reticuloendoplasmic system (RES) contrast agent Endorem was used. Endorem distributes efficiently in tissues where RES is present (e.g. spleen and liver) but is incapable of accessing tumor tissue. To our knowledge this is the first time MRI has been used for evaluation of an animal model of metastatic colorectal cancer.

Our results showed that, when properly optimized, MRI is a powerful method for analyzing the antitumor effects of potential cancer drugs in mice. A large amount of data can be recorded within relatively short imaging sessions. Statistically significant differences between treatment groups were achieved with a small number of animals. Since metastasis is a complex multistep process with several body compartments involved in it, changes in the visceral tumor load should be analyzed at the level of the whole organism. MRI enables this and facilitates, for instance, the accurate timing of treatment in experiments studying specific features, such as pro- and anti-metastatic potencies, of a wide variety of compounds.

As a result, the system described here represents an accurate tool for the study of metastases in mouse as experimental model. The short time needed for each imaging and the low level of anesthesia required, decrease costs and mortality of animals to its minimum. Even if the method is fast, the quality of the images is good enough to fulfill its analytical purposes. This can be seen by the fact that tumors as small as 0,7 mm of diameter can be detected and differed very clearly from other elements present in the tissue. The growth patterns of primary and metastatic tumors are optimal for intervening in each step of the metastatic process separately. This is shown when treating the primary tumor with a therapeutic agent in order to see its effects on primary tumor growth and its consequences on the metastatic process. All in all, our method is a promising tool for the study of metastasis and how experimental treatments can affect their development.

## Materials and Methods

### Ethics Statement

All animal experiments were conducted according to the rules set by the Provincial Government of Southern Finland.

### Cell lines

Human colorectal cancer cell lines Co115, HCT116, SW620, and HT29 were acquired from ATCC (American Type Culture Collection), cultured in the recommended growth media with 10% fetal bovine serum (FBS) and maintained in a humidified atmosphere at 37°C and 5% CO2.

### Oncolytic adenovirus

Ad5-D24-RGD is an oncolytic adenovirus previously described [22]. Briefly, 24bp deletion (“D24”) in the constant region 2 of adenovirus E1A, renders the virus unable to bind retinoblastoma (Rb) and therefore allows virus replication only in Rb/p16 mutant tumor cells. Also, a Arg-Gly-Asp (RGD) aminoacid sequence is inserted in the HI loop of the fiber knob to enhance integrin-dependent entry into cells. Virus was propagated on A549 cells and purified on cesium chloride gradients. The particle concentration was measured at 260 nanometers (nm) and a standard TCID50 test on 293 cells was performed to determine functional units and were assessed to be 1.1x10e12 VP/ml and 2.8x10e10 pfu/ml, respectively.

### Mice

Pathogen-free, 10–11-week-old female SCID (severe combined immunodeficiency) mice were purchased from Taconic (Ejby, Denmark), Charles River (Sulzfeld, Germany), and Harlan (Horst, Holland) and NMRI nude mice from Charles River and Scanbur (Sollentuna, Sweden). Animals were quarantined for 2 weeks. Animals were fed ad libitum and maintained in a HEPA-filtered environment with cages, food, and bedding sterilized by autoclaving.

### MRI

MRI studies were performed with a 4.7 T scanner (PharmaScan, Bruker BioSpin, Ettlingen, Germany) using a 90-mm shielded gradient capable of producing a maximum gradient amplitude of 300 mT/m with an 80-µs rise time. A linear birdcage RF coil with an inner diameter of 38 mm was used. T2-weighted images were acquired using rapid acquisition with relaxation enhancement (RARE) sequence (TR / TEeff  = 3767/ 36 ms, matrix size  = 256×256, Rare Factor  = 8, field-of-view  = 33×33 mm2, 32 slices, slice thickness  = 0.7 mm, number of averages  = 8).

### Spleen-to-liver murine model of metastasized colorectal cancer

The surgical procedure was similar to that previously described [23]. Briefly, 125-day-old SCID mice were anesthetized with ketamine (Ketaminol® 75 mg/kg; Intervet, Boxmeer, Netherlands) / dexmedetomidine (Dexdormitor® 1 mg/kg; Orion Pharm., Espoo, Finland) and the spleen was exteriorized through a left lateral flank incision. Tumors were established by intrasplenic injection of 1x10e6 HT29 cells suspended in 50 µl of serum-free growth media using a 27-gauge needle. The injection site on the spleen was pressed with a cotton stick wet in iodine-polividone solution (Betaine®; Leiras, Helsinki, Finland) in order to destroy extravasated cells and ensure hemostasis. The peritoneum and skin were closed in a single layer with surgical thread. Finally, atipamezole (Antisedan® 1 mg/kg; Orion Pharm., Espoo, Finland) was injected subcutaneously to reverse anesthesia.

For the treatment study, single injection of Ad5-D24-RGD at a dose of 2.5x10e7 VP in 10 ul volume was given into intrasplenic tumors 21 days after cancer cell implantation. Tumor growth (both in the spleen and liver) was followed weekly with MRI for 6 weeks.

### Statistical analyses

Analysis was done with SPSS 15.0 software for Windows. Comparison between mock treated and oncolytic virus treated animals was carried out using t-test for independent samples. A p value less than 0.05 was considered significant.
